# Student Progress Dashboard Versus Legacy Academic Monitoring for Medical Student Support: A Consolidated Framework for Implementation Research-Guided Evaluation

**DOI:** 10.7759/cureus.107976

**Published:** 2026-04-29

**Authors:** Karina R Clemmons, Lindsey Sward, John Spollen, Jasna Vuk, Rebecca Latch, James Graham, Beatrice A Boateng

**Affiliations:** 1 College of Medicine, University of Arkansas for Medical Sciences, Little Rock, USA; 2 Medical Education and Simulation, Med Ed Learning, Anthem, USA

**Keywords:** consolidated framework implementation research, data analytics, faculty advising, precision medical education, student progress dashboard

## Abstract

Background: The 2022 transition of United States Medical Licensure Examination (USMLE) Step 1 to a pass/fail system, coupled with declining national pass rates, has intensified pressure on medical students to succeed on their first attempt and heightened the stakes for Step 2 CK (clinical knowledge) performance. In response to these evolving assessment demands, we developed an innovative Student Progress Dashboard (SPD) to move beyond siloed legacy monitoring. The goal was to apply the tenets of precision education to identify at-risk students and facilitate holistic, timely interventions.

Methods: A mixed-methods evaluation was conducted using the Consolidated Framework for Implementation Research (CFIR). The study integrated: (1) a retrospective-prospective comparison of system functionality, (2) descriptive analytics of system adoption and workflow fidelity among faculty and staff, and (3) a CFIR-guided assessment of stakeholder experiences through surveys and task analyses.

Results: One year post-implementation, usage metrics and survey data indicated that the SPD has been successfully adopted by faculty advisors, course directors, and learning specialists. The dashboard demonstrated significant Relative Advantage (CFIR) over legacy spreadsheets by providing a comprehensive view of the student lifecycle. Results show improved efficiency in identifying students nearing performance thresholds. Course directors reported a clearer view of performance shifts across the curriculum, while learning specialists utilized specific content-area insights to adjust support strategies. Usage averaged 11.5 sessions per month across 25 unique users, with high fidelity to intended workflows.

Conclusion: The SPD is a feasible, scalable tool that addresses the increased pressures of the current medical licensing landscape. By centralizing disparate data into a unified platform, the dashboard enables proactive, individualized support. These findings suggest that while developing such tools is resource-intensive, the benefits, including improved intervention timeliness and enhanced stakeholder productivity, outweigh the investment. Future steps include integrating machine learning for predictive modeling to further refine career and academic advising.

## Introduction

Medical student success is primarily gauged by timely progression, competency attainment, and performance on high-stakes licensing exams. Given this, early identification and support for struggling learners is a top priority for undergraduate medical education programs. To meet this need, educational leaders are increasingly adopting data-driven monitoring to detect emerging problems and coordinate targeted, curriculum-wide, and individualized support [1-3]. The widespread benefit of these tools, however, hinges on faculty and learner acceptance, clear policies regarding data use and privacy, and effective workflows that translate analytics into advising action [4-5]. Without stakeholder buy-in and transparent governance, analytics risk underuse, misinterpretation, or unintended consequences for learners. Assessing attitudes, readiness, and perceived usefulness is therefore essential to responsible implementation.

Against this backdrop, we evaluated our College of Medicine legacy Academic Success Monitoring (ASM) process, which relied on unadjusted first-year (M1) and second-year (M2) exam scores tracked in a spreadsheet and a simple heuristic to flag students at risk of failing the United States Medical Licensure Examination (USMLE) Step 1 on their first attempt based on previous cohorts. The ASM process was implemented in 2016 to proactively identify students at risk of failing USMLE Step 1. The 2022 transition of USMLE Step 1 to pass/fail scoring [6-8], followed by a decline in first-attempt pass rates at our institution and nationally, exposed significant limitations in this manual, retrospective approach. The need for a system that integrates multiple data sources and supports just-in-time, holistic advising became evident. In response, we developed an early warning system, the Student Progress Dashboard (SPD), a scalable longitudinal system that aggregates academic progress, student engagement with academic support resources, and contextual data. The SPD produces real-time risk indicators and enables proactive, individualized advising for students. Launched in 2023, the SPD supports both individual advising (timely interventions) and cohort-level oversight (risk reporting).

The objective of this study was to compare the SPD with the legacy ASM. Our research is guided by three primary aims: (1) Compare Performance: assess the differences in predictive performance and timeliness of risk detection between the ASM and SPD. (2) Assess Implementation Outcomes: evaluate adoption, fidelity, and sustainability of the SPD from the perspectives of college leadership, faculty course directors, academic advisors, and learning specialists. (3) Identify Determinants of Implementation: utilize the Consolidated Framework for Implementation Research (CFIR) [9-10] to identify the specific factors that facilitate or hinder the successful integration of the SPD into the college environment.

These aims identify the contextual factors that enable the transition from tool deployment to sustained institutional practice.

## Materials and methods

The early warning system: SPD 

The SPD was developed by a cross-functional task force including program leadership, faculty advisors, course directors, and learning specialists, and was launched in 2023 in our College of Medicine. Its design was informed by Bloom’s taxonomy, which conceptualizes academic performance as influenced by cognitive, affective, and psychomotor domains [11-12]. This framework guided both the selection of dashboard metrics (Table 1) and the development of domain-aligned interventions intended to support holistic, individualized advising. 

**Table 1:** Metrics Collected in the Database and Visualized in the SPD MCAT: Medical College Admission Test, GPA: grade point average, M1–M4: first through fourth years of medical school, NBME: National Board of Medical Examiners, USMLE: United States Medical Licensing Examination, SPD: Student Progress Dashboard.

Domain	Metrics
Cognitive	Pre-matriculation academic indicators (e.g., MCAT, undergraduate major, and GPA)
	Course and exam performance across M1–M4
	NBME subject exam scores and NBME Comprehensive Basic Science Exam scores
	Objective Structured Clinical Exams
	USMLE STEP 1 and STEP 2 attempts
Affective	Learning Strategies Self-Assessment tool
Psychomotor	Engagement with academic support services (frequency of meetings with a learning specialist, participation in individual or group tutoring, and the development of an individualized study plan)
Other	Name and demographics (including socioeconomic status: rural, first generation, medically underserved)
	Academic risk flag

The cognitive domain captures knowledge acquisition, reasoning, and assessment performance. Indicators include pre-matriculation academic measures (e.g., MCAT (Medical College Admission Test) scores, undergraduate major, and GPA (Grade Point Average)), longitudinal course and exam performance across four years of medical school (M1-M4), NBME subject exams and the Comprehensive Basic Science Exam (CBSE), Objective Structured Clinical Examinations, class rank reports (M1-M3), and all USMLE Step 1 and USMLE Step 2 attempts. These metrics contribute to mastery indicators and predictive risk models that trigger targeted academic supports, such as tutoring, academic coaching, test-taking strategy instruction, and structured study plans.

The affective domain encompasses a student’s motivation, stress levels, and emotional readiness for learning. To monitor this domain, the system tracks specific actionable data points derived from a learning strategies self-assessment tool and the frequency of support service usage. These indicators serve as an early-warning system, triggering personalized student support such as peer tutoring and individualized study plans. These support mechanisms are monitored periodically to evaluate the effectiveness of the interventions and are adjusted accordingly.

The psychomotor domain addresses behavioral and wellness-related factors that influence learning. Indicators include attendance and participation patterns, frequency of meetings with learning specialists, participation in individual or group tutoring, and the presence of an individualized study plan. These data support referrals to wellness services and ongoing coaching related to study habits, sleep hygiene, and other behavioral contributors to academic performance.

In addition to domain-specific indicators, the SPD integrates demographic information, academic performance data, standardized exam scores, engagement logs, and automatically generated academic risk reports. A rules-based algorithm synthesizes these inputs into tiered risk flags accompanied by narrative summaries for advisors. Each risk flag is explicitly linked to prescribed, domain-aligned interventions and standardized workflows for referral, documentation, and longitudinal outcome tracking.

Metric-to-intervention mappings were iteratively refined with end users to ensure clinical and educational validity, minimize false positives, and maintain alignment between identified needs and recommended support pathways. Validation activities included review of historical cases, stakeholder feedback, and alignment checks between risk signals and advising actions, supporting the SPD’s use as a transparent, actionable tool for proactive learner support.

Methods 

Setting

This study was conducted at a single U.S. allopathic medical school (UAMS). The project was reviewed by the Institutional Review Board and determined to be exempt from human subjects review (UAMS IRB #298975).

Inclusion and Exclusion Criteria

This implementation evaluation included institutional stakeholders involved in academic support and curriculum oversight. Participants included faculty advisors, learning specialists, course directors, and academic affairs leaders. Students were excluded from participation and were not surveyed or interviewed. This study represents an initial CFIR-informed implementation phase focused on adoption and workflow integration among faculty advisors. CFIR includes constructs relevant to end-user experience, which in this case are faculty, not students.

Study Design

This study employed a mixed-methods, multi-component evaluation to compare the SPD with the legacy ASM process. The evaluation integrated (1) a retrospective-prospective comparison of student-level risk flagging performance and timeliness, (2) descriptive analyses of system adoption and workflow fidelity, and (3) a CFIR-guided implementation assessment of stakeholder experiences. We embedded an implementation science approach at the outset to examine not only whether the SPD was associated with improved identification and timing of academic risk detection, but also how the system was adopted, used, and sustained in routine advising practice. CFIR-informed study questions, measurement, and interpretation of stakeholder-facing outcomes enable linking usability and perception findings to actionable implementation strategies. Outcomes were assessed for the SPD only, as the legacy ASM process was no longer in active use and had not been systematically evaluated.

Stakeholder Surveys

We developed a CFIR-informed stakeholder survey to assess perceptions of the SPD’s usability, fit within existing workflows, and implementation processes (Appendices). Survey items were mapped a priori to the CFIR domains: Intervention Characteristics, Outer Setting, Inner Setting, Characteristics of Individuals, and Implementation Process. The survey included role-based administrative items, frequency of dashboard use, and device access, followed by 5-point Likert-scale items (1 = strongly disagree to 5 = strongly agree) assessing accessibility, timeliness, workflow integration, leadership support, confidence in interpretation, perceived usefulness, and training adequacy. Open-ended questions were included to elicit perceived barriers and facilitators to use, training needs, and examples of dashboard-driven advising actions.

The instrument was developed by the implementation team with input from advisors and learning specialists and was intended for descriptive implementation evaluation rather than psychometric validation. Survey invitations were distributed electronically to faculty advisors, learning specialists, course directors, and academic affairs leaders, with three reminders sent over a 90-day period.

Statistical Analysis

Quantitative data were analyzed descriptively. Usage metrics (e.g., number of unique users, frequency of dashboard access, and page-level activity) were summarized using counts, proportions, and measures of central tendency. Survey responses to Likert-scale items were summarized using frequencies, percentages, and descriptive statistics (means and standard deviations where appropriate). No inferential statistical tests were planned or conducted, as the evaluation aimed to characterize implementation outcomes rather than test hypotheses or compare groups.

Qualitative data from open-ended survey responses and observational notes were analyzed using a rapid content analysis approach. Two members of the implementation team independently reviewed responses to identify recurring concepts related to usability, workflow integration, training needs, and perceived value. Themes were organized and interpreted using the CFIR, which guided integration of quantitative and qualitative findings across the five implementation domains.

## Results

The evaluation of the SPD demonstrated a successful transition from a manual, reactive monitoring process to an automated, predictive system integrated into the academic support infrastructure. The findings are structured to provide a comprehensive view of this transition: first, a functional comparison between the legacy ASM spreadsheet and the SPD; second, an analysis of system adoption and usage metrics; and third, a CFIR-guided assessment of stakeholder perceptions across the five implementation domains. Collectively, the results indicate that the SPD not only improved the technical efficiency of risk detection but also achieved high levels of "relative advantage" and workflow compatibility, effectively shifting the institutional culture toward proactive, data-informed student advising.

System transition and functional comparison

To contextualize the transition from the legacy ASM process to the SPD, the two systems were compared across key functional domains. This comparison was mapped to the CFIR to identify the theoretical drivers of adoption. Table 2 presents a side-by-side comparison of the ASM spreadsheet and the current SPD, mapped to relevant CFIR constructs. It illustrates that the SPD was intentionally designed to overcome the limitations of the legacy spreadsheet by improving accessibility, visualization, data integration, and actionable insights.

**Table 2:** System Adoption, Usage Metrics, and Workflow Fidelity for the Student Progress Dashboard SPD: Student Progress Dashboard, M1–M2: first and second years of medical school, USMLE: United States Medical Licensing Examination, ASM: Academic Success Monitoring.

Domain	Metric/Observation	Summary
System adoption and usage	Unique users	~25 unique users accessed the SPD in the first year.
	Average monthly views	Users viewed the dashboard an average of 11.5 times per month.
	Most frequently accessed pages	M1–M2 grades pages were the most frequently accessed.
	Other commonly accessed pages	Individual student profiles
		Lists of students contacted to meet with a learning specialist
		Learning specialist activity page (meeting frequency, individualized plans)
		USMLE Step 1 at risk report
	Device and access patterns	Primarily desktop and laptop use; mobile/tablet use less common (from survey).
Workflow fidelity and task analyses	Integration into the ASM workflow	SPD used as the “single source of truth” during post-exam reviews.
	Core tasks performed consistently	Filtering for at-risk status; mapping support needs; reviewing longitudinal performance.
	Efficiency gains	Stakeholders reported reduced time to identify at-risk cohorts compared with the legacy spreadsheet.
	Workarounds/deviations	Early implementation workarounds (e.g., navigating filters) decreased as users gained proficiency.
	New capabilities enabled	Rapid identification of students nearing policy thresholds; tracking individualized study plans; flagging students for board prep support.

System adoption and usage

One year after implementation, usage metrics largely reflected stakeholder survey responses. Metrics such as number of accesses, time spent, frequency of use, and user roles were reviewed. The most frequently accessed pages of the SPD were the M1-M2 grades pages, which align with their central role in ASM committee discussions after each exam. Approximately 25 unique users have viewed the dashboard an average of 11.5 times per month since launch. Other commonly accessed pages included individual student profiles, lists of students who had been contacted to meet with a learning specialist, the learning specialists’ page documenting frequency of meetings and individualized learning plans, and the USMLE Step 1 at-risk student report.

Following this functional comparison, we next examined system adoption patterns and workflow integration during the first year of implementation. Key usage metrics and workflow fidelity observations are summarized in Table 3. 

**Table 3:** Side-by-Side Comparison With ASM Spreadsheet ASM: Academic Success Monitoring, SPD: Student Progress Dashboard, CFIR: Consolidated Framework for Implementation Research, M1–M2: first and second years of medical school, M1–M4: first through fourth years of medical school.

Feature	ASM Spreadsheet (Legacy)	Student Progress Dashboard (SPD)	Relevant CFIR Construct(s)
Accessibility	Localized; restricted to single-terminal access.	Cloud-based; multi-device, remote access.	Relative advantage; adaptability
Visual analytics	Static; manual data entry; no visualization.	Automated; interactive visualizations across domains.	Design quality & packaging
Data scope	Restricted to M1–M2 years.	Longitudinal (M1–M4); includes leaves of absence.	Outer setting (Student needs)
Integrated data	Absent; no student self-assessment data.	Integration of learning strategies self-assessment tool results and at-risk flags	Relative advantage; Student needs

Workflow fidelity and task analyses

The SPD has successfully integrated into the formal ASM committee workflow, serving as a "single source of truth" during post-exam reviews. Users consistently performed core tasks, including filtering for at-risk status and mapping support needs. The transition from a manual spreadsheet to a guided, coordinator-led visual discussion aligns with the intended workflow design. While formal time-motion data were not collected, stakeholder feedback indicates a significant reduction in the time required to identify at-risk cohorts compared to the legacy ASM spreadsheet. Observed "workarounds" or deviations were limited to the initial implementation phase (e.g., navigating complex filters). These decreased as users gained "Individual Stage of Change" proficiency (CFIR construct).

Qualitative observations indicate that the SPD has increased efficiency and reduced manual effort compared with the legacy ASM spreadsheet. Tasks that previously required scrolling through multiple tabs or manual calculations are now streamlined through interactive filtering and visualizations. The SPD also enables new capabilities, such as quickly identifying students nearing policy thresholds for enhanced academic support, tracking individualized study plans, and flagging students for additional board exam preparation resources. Users reported that these features facilitate timelier, more targeted advising interventions.

CFIR guided survey: perceptions of dashboard 

Of the 80 invited stakeholders, 23 completed the survey, yielding a 29% response rate. Respondents included faculty advisors, learning specialists, course directors, academic affairs leaders, and other educational roles. Most respondents (19/23; 83%) reported using the dashboard in the past year, while four (17%) had not.

Among respondents who reported that they had not yet utilized the dashboard, the primary barrier identified was a perceived need for additional training. Specific qualitative feedback highlighted diverse reasons for non-use: one respondent noted a preference for mobile-responsive access, while another cited a lack of time and perceived low relevance, as they managed a small student cohort. These responses suggest that while the SPD offers high functionality, adoption is still influenced by individual workflow demands and the "compatibility" (CFIR) of the tool with specific user roles.

CFIR domains 

To contextualize stakeholder experiences with the SPD, Table 4 provides a domain-level summary of CFIR-guided survey responses and qualitative feedback.

**Table 4:** CFIR Guided Survey Findings: Quantitative Results and Qualitative Themes SPD: Student Progress Dashboard, CFIR: Consolidated Framework for Implementation Research.

CFIR Domain	Quantitative Findings	Qualitative Themes/Illustrative Comments
Intervention characteristics	100% agreed/strongly agreed that the dashboard was easily accessible and updated in a timely manner.	Centralized view of student progress is highly valued.
		Visualizations improve efficiency compared with a spreadsheet.
		“Transformational” for advising; “one stop shop.”
Outer setting	Supports student preparation for exams and Step 1 (78%).	Dashboard use driven by student needs.
	Provides timely information (65%).	Helps identify struggling students early.
	Aligns with academic support policies (70%).	Enables individualized advising based on performance trends.
	Supports advisor workflow.	“Invaluable objective collateral information.”
Inner setting	n = 18	Few organizational barriers.
	Easy access when needed (83.3%).	Practical issues: navigating between systems, locating updated Step 1 data.
	Fits existing workflows (94.4%).	
	Leadership encourages use (83.3%).	Barriers relate to system integration, not culture or support.
Characteristics of individuals	n = 18	SPD is intuitive and user-friendly.
	Confident interpreting indicators (83.3%).	Training needs to focus on refreshers and advanced features.
	Dashboard improves student outcomes (94.4%).	
	Concerns about data use (16.7%).	Confidence increases with regular use.
Implementation process	n = 18	Users want brief videos, refreshers, clearer documentation.
	Support team responsive (83.3%).	Nonusers cite lack of onboarding and unclear role relevance.
	Training adequate (77.8%).	Nonuse driven by limited training, not negative perceptions.
	Clear directions available (66.7%).	
Overall value	n = 17	A few improvement suggestions.
	Nearly all agreed that SPD enhanced the ability to support students.	Desired enhancements: automated reports, expanded data sources.
	Only one neutral response.	Interest in optimization rather than redesign.

Intervention Characteristics

Stakeholders generally rated the dashboard positively in terms of accessibility and timeliness. For dashboard accessibility, as seen in Table 4, all respondents (100%) agreed or strongly agreed that the dashboard was easily accessible online and updated in a timely manner, indicating that technical access and data refresh did not present major barriers to use. Open-ended feedback in Table 5 reinforced these findings. 

**Table 5:** Qualitative Comments by SPD Users SPD: Student Progress Dashboard.

Qualitative Comments by SPD Users
"It was nice to be able to see one student’s progress and history in one place."
"Having all this data at our fingertips has been transformational in the way we provide advising to students. It has allowed us to see trends that weren't as obvious before. The ease of access for advisors has also greatly improved."
"This is a substantial improvement. I can see the entire academic performance of an individual student over the first two years in a very easily interpretable graphic."
"This dashboard has revolutionized student advising and provides such an easy one-stop-shop to check on my students periodically. I absolutely love it and use it consistently."

Together, these findings suggest that the dashboard’s design and accessibility were perceived as clear improvements over the legacy spreadsheet and supported efficient review of student progress.

Outer Setting

Responses to outer setting items indicated that stakeholders perceived the dashboard as well aligned with external expectations and student needs. Most respondents agreed or strongly agreed that the dashboard supports student preparation for course exams and USMLE Step 1 (78%), provides timely information (65%), and aligns with institutional academic support policies (70%).

Qualitative responses emphasized that student needs were the primary driver of dashboard use, particularly for identifying students at risk, contextualizing performance, and tailoring student support. Advisors and learning specialists frequently check the dashboard to identify students struggling in coursework or at risk of failing exams, enabling timely interventions. Respondents noted that reviewing performance data helps them individualize advice or support strategies based on individual student strengths, weaknesses, or personal circumstances.

One stakeholder commented, "The dashboard has significantly improved my ability to assess the students’ academic functioning when making decisions. It provides invaluable objective collateral information. Being able to identify students at risk of failing Step 1 in advance has also been very helpful. With this information, we are able to strongly encourage them to utilize learning resources and follow up with them closely during their dedicated time to provide emotional support." This response highlights that student-specific needs are the main driver of dashboard engagement and that the system facilitates proactive, data-informed advising.

Inner Setting

Among respondents who answered inner setting items (n = 18), perceptions of organizational fit and support were largely favorable. Most stakeholders agreed or strongly agreed that they had easy access to the dashboard when needed (83.3%), that the dashboard fit well within existing workflows for advising, teaching, and program oversight (94.4%), and that leadership actively encouraged dashboard use in student support activities (83.3%).

Open-ended responses regarding organizational barriers indicated few substantive impediments to dashboard use. Several respondents explicitly reported no barriers or were unsure of any organizational challenges. The most commonly cited issues were practical rather than structural. These included difficulty navigating between the SPD and other institutional systems, such as the curriculum database, and occasional delays or uncertainty regarding the availability of updated USMLE Step 1 information. These comments suggest that the remaining barriers relate primarily to system integration and user familiarity, rather than to workflow misalignment or a lack of institutional support.

Individual Characteristics

Respondents reported high levels of confidence and perceived usefulness related to the SPD. Among those who responded to these items (n = 18), most agreed or strongly agreed that they were confident in interpreting SPD indicators and color coding (83.3%) and that the SPD was useful for improving student outcomes (94.4%). In contrast, relatively few respondents expressed concerns about how SPD data might be used or interpreted. Only 16.7% agreed or strongly agreed that they had such concerns, while the majority disagreed or remained neutral, suggesting generally high trust in the SPD’s use for student support.

Open-ended responses regarding training and support needs reinforced these findings. Many respondents indicated that the SPD was intuitive and user-friendly, with several noting that increased familiarity through regular use was sufficient to build confidence. However, multiple respondents suggested that brief training or refresher sessions would be beneficial, particularly for new users or to highlight less obvious features. Suggestions included overviews of SPD functionality and guidance on interpreting trends over time, rather than fundamental usability training.

Implementation Process

Among respondents who reported using the SPD in the prior year (n = 18), perceptions of the implementation process were generally positive. Most agreed or strongly agreed that the SPD support team was responsive to feedback (83.3%) and that the training they received adequately prepared them to use the dashboard (77.8%). Agreement was somewhat lower for the availability of clear directions describing how and when to use the dashboard (66.7%), suggesting variability in how consistently implementation guidance was perceived across users.

Open-ended responses regarding implementation improvements revealed meaningful differences between SPD users and non-users. SPD users most frequently suggested incremental refinements, such as brief training videos, refresher sessions, and clearer documentation of advanced features and reporting capabilities. Several users noted that they did not recall receiving formal training but were able to learn the system through use, indicating that training gaps were not prohibitive but could be addressed to improve efficiency and confidence. In contrast, non-users’ comments emphasized lack of familiarity with the SPD’s purpose, uncertainty about its relevance to their role, and the need for initial training or clearer integration into existing systems and workflows. These responses suggest that non-use was driven more by limited onboarding and role alignment than by negative perceptions of the SPD itself.

Overall Assessment of Dashboard Value

Among respondents who answered the overall value item (n = 17), nearly all indicated that the SPD enhanced their ability to support students. Only one respondent provided a neutral response. These findings indicate a strong perceived overall value among stakeholders who engaged with the SPD.

Open-ended responses regarding improvements to further increase the dashboard’s value were limited and focused primarily on incremental enhancements. Several respondents indicated that no improvements were needed at this time. Suggested enhancements included automated reports for course directors that identify at-risk students at the start of new courses and the expansion of SPD content to include additional performance data, such as clerkship evaluations and future scheduling information. These suggestions reflect interest in extending the SPD’s functionality rather than concerns about its core purpose or usability. Table 6 summarizes the key CFIR findings and implications for implementation. 

**Table 6:** CFIR Summary of Student Progress Dashboard Implementation Findings CFIR: Consolidated Framework for Implementation Research, USMLE: United States Medical Licensing Examination.

CFIR Domain	Key Quantitative Findings	Key Qualitative Findings/Illustrative Themes	Implications for Implementation
Intervention characteristics	High agreement that the dashboard was accessible, timely, and supported student preparation. The majority agreed that the data were presented in a usable and actionable format.	Stakeholders valued the ability to view a student’s academic history and progress longitudinally in one place. Visualizations and centralized data improved efficiency compared with the spreadsheet.	The dashboard demonstrated a clear relative advantage and strong design quality over the legacy spreadsheet, supporting adoption.
Outer setting	Most respondents agreed that the dashboard supported early intervention for exams and accommodated individualized student timelines.	Use of the dashboard was driven by student needs (e.g., struggling learners, USMLE Step 1 preparation). Respondents described using objective data to guide advising and support decisions.	External pressures (USMLE Step 1 performance, learner support expectations) aligned well with dashboard functionality.
Inner setting	Among respondents (n = 18), most agreed or strongly agreed that the dashboard was easily accessible (83.3%), fit within existing workflows (94.4%), and was supported by leadership (83.3%).	Few organizational barriers were identified. Comments focused on practical issues (e.g., navigating between systems, locating updated exam data) rather than cultural or structural resistance.	Strong organizational readiness and leadership engagement facilitated early adoption and routine use.
Characteristics of individuals	High confidence in interpreting dashboard indicators (83.3%) and strong belief that the dashboard improves student outcomes (94.4%). Few respondents reported concerns about data use (16.7%).	Respondents described the dashboard as intuitive and user-friendly. Training needs focused on refreshers, trend interpretation, and awareness of advanced features rather than basic functionality.	Individual knowledge, skills, and beliefs supported implementation; limited concerns suggest trust in dashboard governance.
Implementation process	Among dashboard users (n = 18), most agreed that training was adequate (77.8%) and that the support team was responsive (83.3%); agreement was lower for clarity of directions (66.7%).	Users suggested brief training videos, refresher sessions, and improved documentation. Non-users cited a lack of onboarding, unclear relevance to their role, and a need for initial training.	Implementation was generally effective, with opportunities to strengthen onboarding and role-specific guidance to improve uptake.
Overall value	Among respondents who answered the item (n = 17), nearly all agreed or strongly agreed that the dashboard enhanced their ability to identify and support at-risk students.	Few improvement suggestions were offered; those provided focused on optimization (automated reports, expanded data sources) rather than redesign.	Strong perceived value supports sustainability and continued investment in the dashboard.

## Discussion

Implementation success and stakeholder acceptance

Our evaluation demonstrates that the SPD was successfully implemented and largely well received by stakeholders. A high proportion of survey respondents (83%) reported using the dashboard, indicating strong adoption among faculty advisors, learning specialists, course directors, and academic affairs leaders. Users reported high confidence in accessing the dashboard, interpreting student progress indicators, and applying insights to support at-risk students. Open-ended responses reinforced these findings, highlighting the value of having integrated, actionable student data in one accessible platform (Table 4). These results align with previous studies that indicate that dashboards can be used to identify programmatic and assessment gaps and support continuous quality improvement in medical education and programmatic assessment [2,13-14]. The results also align with CFIR constructs of Inner Setting (workflow fit) and Characteristics of Individuals (knowledge, self-efficacy) (Table 5), suggesting that organizational readiness and user competence facilitated early adoption.

Advantages over the legacy system

A comparative analysis of the SPD against the legacy ASM spreadsheet shows significant gains in functionality, usability, and operational efficiency. Stakeholder feedback suggests that the SPD’s primary Relative Advantage (CFIR) lies in its ability to consolidate the longitudinal student lifecycle and integrate disparate data sources into a unified visualization (Table 5). Quantitative frequency metrics and task analyses confirm that the dashboard has successfully transitioned from a passive record-keeping tool to an active decision-support system. By reducing manual data manipulation, the SPD facilitates the timely identification of students nearing critical performance thresholds. Qualitative data from open-ended comments further illustrate that these technical improvements have shifted faculty and staff behaviors from a reactive stance to a more proactive intervention model, allowing support to be deployed before academic failure occurs (Table 4). Crucially, the integration of at-risk flags allows the SPD to provide a more holistic view of student readiness than was possible with the grade-centric legacy spreadsheet.

Implementation science process and support

Survey responses indicate that the implementation process, including training and support, was generally effective (Table 4). Most users agreed that training prepared them to use the dashboard and that the support team was responsive to feedback. Some non-users or infrequent users noted the need for additional guidance and onboarding, highlighting opportunities for improvement. This aligns with CFIR Implementation Process constructs and learning analytics in medical education [15-16], emphasizing the importance of continuous feedback, responsive support, and clear guidance to enhance adoption.

Remaining challenges and opportunities

Despite positive findings, several areas for refinement emerged. Open-ended responses suggested a desire for targeted onboarding, refresher sessions, and quick-reference guides. Some stakeholders noted variability in engagement due to competing priorities or limited familiarity with dashboard features. Additionally, predictive modeling and longitudinal tracking could be enhanced further to support more nuanced risk identification and intervention planning. Addressing these areas could further improve usability and sustainability, consistent with CFIR constructs of Intervention Complexity and Adaptability.

Implications for practice

The SPD demonstrates the value of integrating multiple data sources into a centralized, accessible platform for early warning and student support. Implementation science principles, particularly CFIR, proved useful for evaluating not only system effectiveness but also adoption, workflow integration, and stakeholder perceptions [15]. Other medical schools or educational programs considering similar interventions can use these findings to guide system design, implementation planning, and training strategies.

Limitations

This study is limited by the small number of respondents (n=23), which may affect generalizability. Survey responses reflect self-reported perceptions rather than direct measures of student outcomes, although usage metrics partially corroborate reported engagement. Limitations of the SPD include ensuring the accuracy of data entry and continued faculty development in the use of data analytics for advising. Future research could link dashboard use to measurable student performance or retention outcomes and explore long-term sustainability.

## Conclusions

As learning analytics becomes more important in medical education, the SPD represents a shift from reactive monitoring to a proactive, data-driven support model. By centralizing disparate data into a unified, visual interface, the SPD has improved workflow efficiency and the timeliness of interventions for at-risk students. This evaluation, guided by the CFIR framework, demonstrates that the success of such an intervention hinges not only on technical design but also on institutional leadership, stakeholder engagement, and a seamless fit within existing academic workflows. While this study reflects implementation at a single institution, the core findings suggest that the SPD is a scalable and feasible model for other medical schools seeking to modernize their student success infrastructure by using the data already available to them.

Future iterations of the dashboard aim to transition from descriptive analytics toward precision medical education through the integration of machine learning and regression-based algorithms. By using longitudinal data, these predictive models will allow for individualized performance forecasting, enabling earlier support for students prior to high-stakes licensure examinations. Furthermore, expanding dashboard access to career and physician advisors would provide a comprehensive data foundation for specialty counseling. By using predictive simulations of student outcomes, including clerkship performance and residency matching likelihood, advisors could offer personalized, scenario-based guidance throughout the medical school lifecycle.

## Figures and Tables

**Figure 1:**
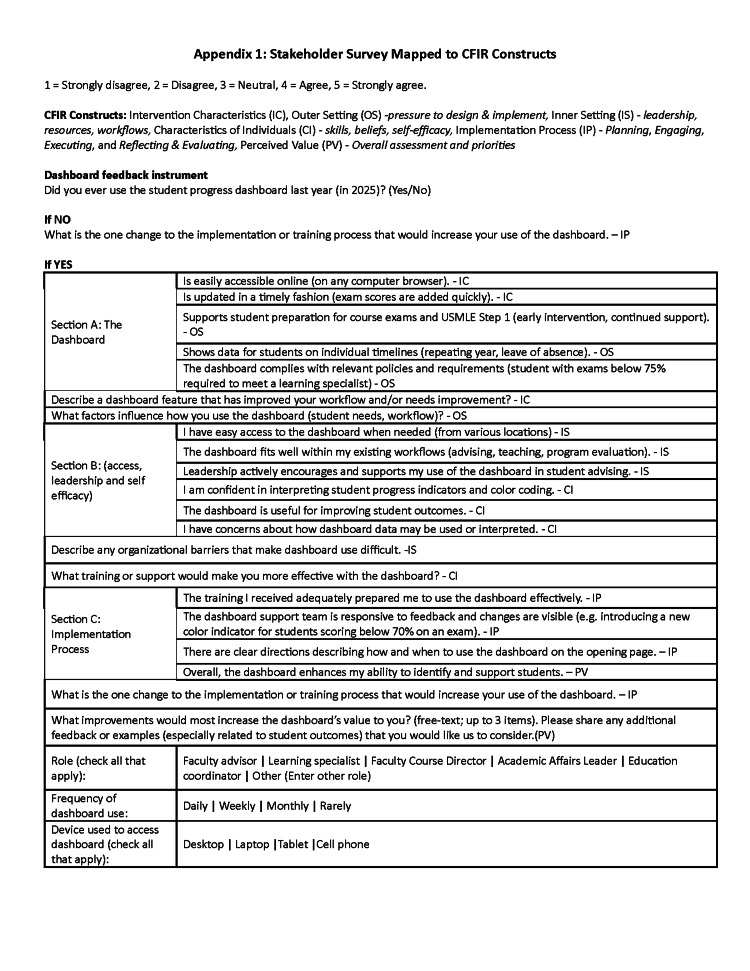
Survey Section for Individuals Who Have Accessed the Dashboard CFIR: Consolidated Framework for Implementation Research.
